# Successful appendiceal incision by endoscopic submucosal dissection to allow endoscopic removal of an encapsulated fecalith

**DOI:** 10.1055/a-2191-2475

**Published:** 2023-11-20

**Authors:** Qingyu Zeng, Zhang Tao, Jie Liu, Kong Tao, Xu Shan, Ya Lan Chen

**Affiliations:** 1 74647 Department of Gastroenterology, The Affiliated Hospital, Southwest Medical University, Luzhou, China; 2 Department of Gastroenterology, Nanchong Central Hospital, The Second Clinical Medical College, North Sichuan Medical College, Nanchong, China; 3 Department of Gastroenterology, Nanchong Central Hospital, The Second Clinical Medical College, North Sichuan Medical College, Nanchong, China

A 53-year-old man was admitted to our gastroenterology department for investigation of a colonic lump. Ultrasonic colonoscopy revealed a lump (about 0.8 cm) blocking the opening of the appendix and an encapsulated fecalith between the submucosa and the muscularis propria ( Fig. 1 and Fig. 2 **a** ). Removal of the encapsulated fecalith was difficult using only an endoscope. Therefore, the mucosa and submucosa were incised in the middle of the lump via endoscopic submucosal dissection (ESD); however, a scar was detected during the mucosal and submucosal incision ( Video 1 ). The scar was incised with a DualKnife, subsequently revealing a hard yellow encapsulated fecalith ( Fig. 2 **b** ). The lump had originated from inflammatory hyperplasia caused by the encapsulated fecalith. The encapsulated fecalith (about 1×1 cm) was removed using an endoscope ( Fig. 3 ). Some bleeding at the incision site was detected endoscopically, and hemostatic clips were applied to stop this. Appendicography showed no residual encapsulated fecalith. The patient experienced slight abdominal distension at the end of the therapeutic endoscopy. He was discharged from the hospital 5 days later with no symptoms.

**Fig. 1:**
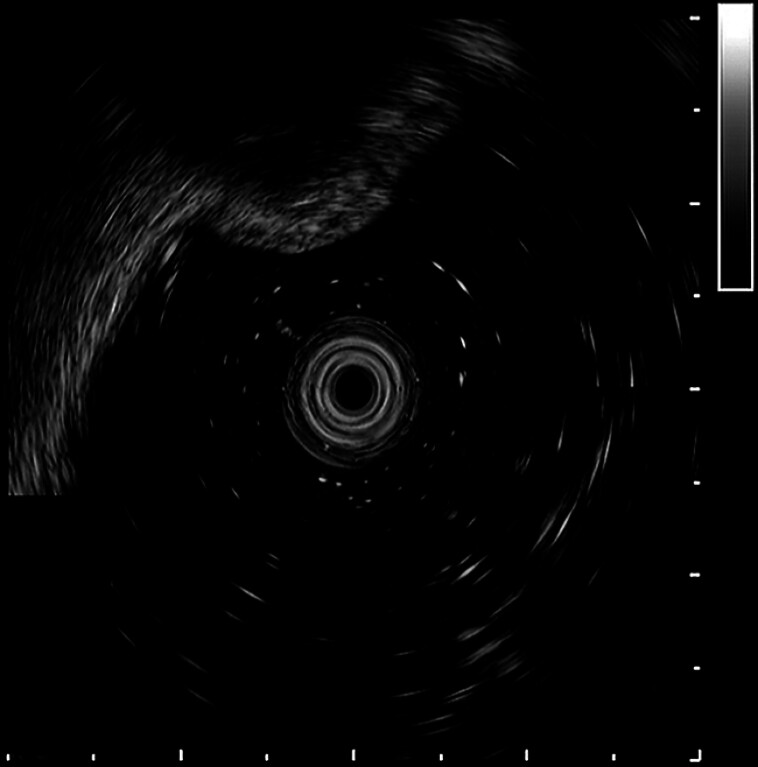
Ultrasonographic image showing the encapsulated fecalith between the submucosa and muscularis propria.

**Fig. 2:**
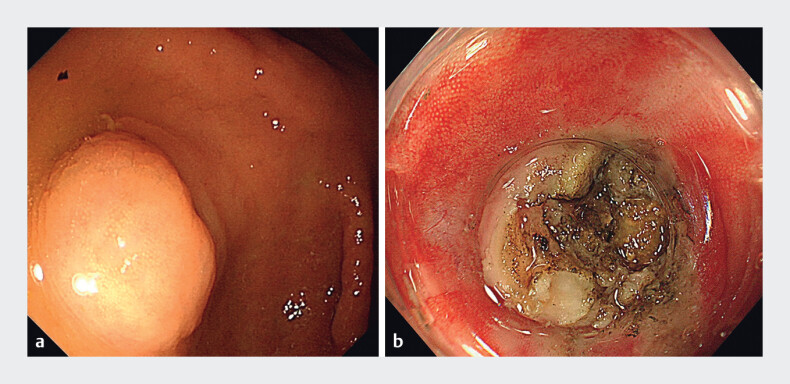
Endoscopic images showing: **a** the lump (about 0.8 cm); **b** the view following incision of the mucosa, submucosa, and the scar.

The lump at the opening of the appendix was incised via endoscopic submucosal dissection allowing the encapsulated fecalith to be easily removed endoscopically.Video 1

**Fig. 3:**
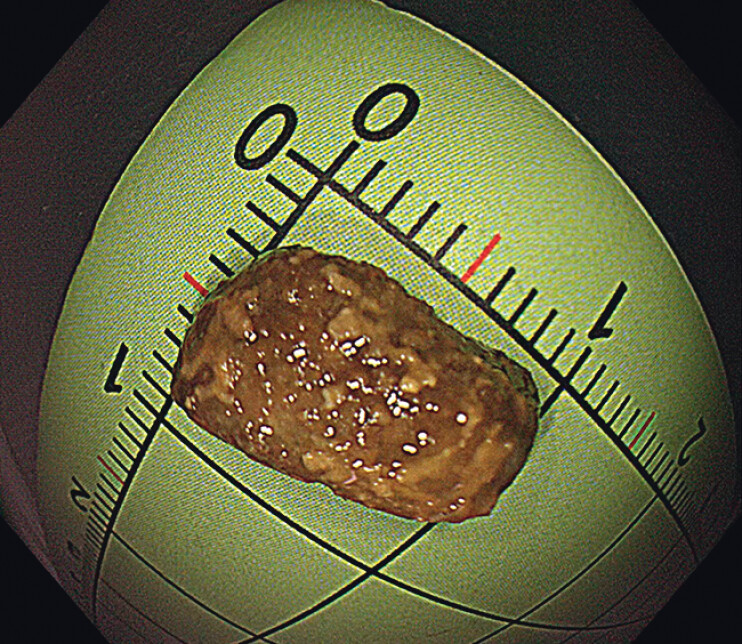
Photograph of the encapsulated fecalith (about 1 × 1 cm) after its removal.

A lump blocking the appendix, which can be detected by imaging, suggests the presence of a fecalith. The lump should be carefully identified, and cutting of the lump should be carefully avoided. Fecaliths cause appendicitis by closing the appendiceal cavity 1 2 . The presence of an appendicolith is also an independent risk factor for appendiceal perforation and gangrene 3 . Incision of the appendix via ESD exposes the fecalith and facilitates its removal. ESD is a new and feasible treatment for more complicated cases of encapsulated fecalith, such as those associated with inflammatory hyperplasia.

Endoscopy_UCTN_Code_TTT_1AQ_2AF

**Correction**

**Correction: Successful appendiceal incision by endoscopic submucosal dissection to allow endoscopic removal of an encapsulated fecalith** Qingyu Zeng, Zhang Tao, Jie Liu et al. Successful appendiceal incision by endoscopic submucosal dissection to allow endoscopic removal of an encapsulated fecalith. Endoscopy 2023; 55: E1156–E1157, doi:10.1055/a-2191-2475 In the above-mentioned article the institution affiliation 1 has been corrected. Correct is: Department of Gastroenterology, The Affiliated Hospital, Southwest Medical University, Luzhou, China. This was corrected in the online version on November 11, 2024.

## Notes

Endoscopy E-Videos https://eref.thieme.de/e-videos
*E-Videos* is an open access online section of the journal *Endoscopy* , reporting on interesting cases and new techniques in gastroenterological endoscopy. All papers include a high-quality video and are published with a Creative Commons CC-BY license. Endoscopy E-Videos qualify for HINARI discounts and waivers and eligibility is automatically checked during the submission process. We grant 100% waivers to articles whose corresponding authors are based in Group A countries and 50% waivers to those who are based in Group B countries as classified by Research4Life (see: https://www.research4life.org/access/eligibility/ ). This section has its own submission website at https://mc.manuscriptcentral.com/e-videos .

## Notes

Correction **Correction: Successful appendiceal incision by endoscopic submucosal dissection to allow endoscopic removal of an encapsulated fecalith** Qingyu Zeng, Zhang Tao, Jie Liu et al. Successful appendiceal incision by endoscopic submucosal dissection to allow endoscopic removal of an encapsulated fecalith. Endoscopy 2023; 55: E1156–E1157, doi:10.1055/a-2191-2475 In the above-mentioned article the institution affiliation 1 has been corrected. Correct is: Department of Gastroenterology, The Affiliated Hospital, Southwest Medical University, Luzhou, China. This was corrected in the online version on November 11, 2024.
