# Correction: Successful appendiceal incision by endoscopic submucosal dissection to allow endoscopic removal of an encapsulated fecalith

**DOI:** 10.1055/a-2464-6402

**Published:** 2024-11-12

**Authors:** Qingyu Zeng, Zhang Tao, Jie Liu, Kong Tao, Xu Shan, Ya Lan Chen

**Affiliations:** 1 74647 Department of Gastroenterology, The Affiliated Hospital, Southwest Medical University, Luzhou, China; 2 Department of Gastroenterology, Nanchong Central Hospital, The Second Clinical Medical College, North Sichuan Medical College, Nanchong, China; 3 Department of Gastroenterology, Nanchong Central Hospital, The Second Clinical Medical College, North Sichuan Medical College, Nanchong, China



In the above-mentioned article the institution affiliation 1 has been corrected. Correct is: Department of Gastroenterology, The Affiliated Hospital, Southwest Medical University, Luzhou, China. This was corrected in the online version on November 11, 2024.

